# The Endocannabinoid System as a Target in Cancer Diseases: Are We There Yet?

**DOI:** 10.3389/fphar.2019.00339

**Published:** 2019-04-05

**Authors:** Estefanía Moreno, Milena Cavic, Ana Krivokuca, Vicent Casadó, Enric Canela

**Affiliations:** ^1^Department of Biochemistry and Molecular Biomedicine, Faculty of Biology, Institute of Biomedicine (IBUB), University of Barcelona, Barcelona, Spain; ^2^Centro de Investigación Biomédica en Red sobre Enfermedades Neurodegenerativas (CIBERNED), Madrid, Spain; ^3^Department of Experimental Oncology, Institute for Oncology and Radiology of Serbia, Belgrade, Serbia

**Keywords:** endocannabinoids, G protein-coupled receptor, cannabinoid CB1 receptor, cannabinoid CB2 receptor, ethical issues, marijuana legalization, receptor heteromer

## Abstract

The endocannabinoid system (ECS) has been placed in the anti-cancer spotlight in the last decade. The immense data load published on its dual role in both tumorigenesis and inhibition of tumor growth and metastatic spread has transformed the cannabinoid receptors CB1 (CB1R) and CB2 (CB2R), and other members of the endocannabinoid-like system, into attractive new targets for the treatment of various cancer subtypes. Although the clinical use of cannabinoids has been extensively documented in the palliative setting, clinical trials on their application as anti-cancer drugs are still ongoing. As drug repurposing is significantly faster and more economical than *de novo* introduction of a new drug into the clinic, there is hope that the existing pharmacokinetic and safety data on the ECS ligands will contribute to their successful translation into oncological healthcare. CB1R and CB2R are members of a large family of membrane proteins called G protein-coupled receptors (GPCR). GPCRs can form homodimers, heterodimers and higher order oligomers with other GPCRs or non-GPCRs. Currently, several CB1R and CB2R-containing heteromers have been reported and, in cancer cells, CB2R form heteromers with the G protein-coupled chemokine receptor CXCR4, the G protein-coupled receptor 55 (GPR55) and the tyrosine kinase receptor (TKR) human V-Erb-B2 Avian Erythroblastic Leukemia Viral Oncogene Homolog 2 (HER2). These protein complexes possess unique pharmacological and signaling properties, and their modulation might affect the antitumoral activity of the ECS. This review will explore the potential of the endocannabinoid network in the anti-cancer setting as well as the clinical and ethical pitfalls behind it, and will develop on the value of cannabinoid receptor heteromers as potential new targets for anti-cancer therapies and as prognostic biomarkers.

## Introduction

**FIGURE 1:**
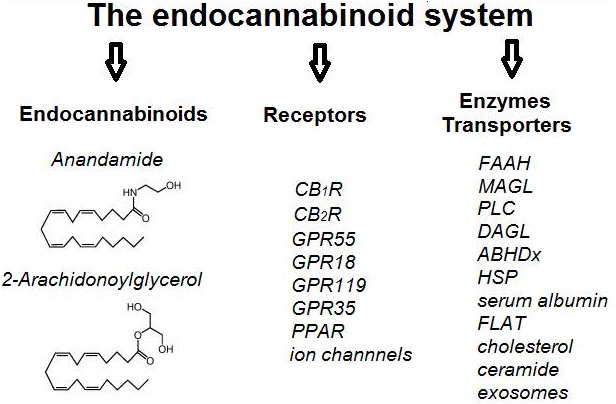
A schematic representation of the main components of the endocannabinoid system (ECS). CBR – cannabinoid receptor, GPR – G protein-coupled receptor, PPAR – peroxisome-proliferator-activated receptors, FAAH – Fatty acid amide hydrolase, MAGL – Monoacylglycerol lipase, PLC – Phospholipase C, DAGL – Diacylglycerol lipase, ABHDx – Alpha beta hydrolase domain proteins, HSP – heat shock proteins, FLAT – FAAH-like anandamide transporter.

The term endocannabinoid system (ECS) refers to a complex network of cannabinoid receptors, endocannabinoid ligands, the enzymatic machinery that drives their biosynthesis, degradation, transport and all cells and neurological pathways that involve endocannabinoid signaling (Figure 1). It is implicated in the control of the most vital processes thus creating homeostasis within the organism, which explains its ambiguous role in tumorigenesis and suppression of tumors.

The ECS components were gradually described in the late 1980s and early 1990s during the search for the pharmacological targets of Δ9-tetrahydrocannabinol (THC) isolated from the plant *Cannabis sativa* (marijuana) (Devane et al., 1988; Matsuda et al., 1990). The discovery was a classical success story that went much beyond the intended purpose of providing mechanistic proof for the psychotropic effects of cannabinoids. As cannabinoid consumption was alarmingly rising especially in more developed countries, a lot of funding was invested in projects trying to decipher the exact mode of its action in order to strengthen the publics “no” vote for marijuana legalization (Thomas, 2001). However, the fact that marijuana was also invaluable in easing the symptoms of many other conditions (nausea, pain, motor dysfunctions, cardiovascular, gastrointestinal and reproductive problems etc.) could not be ignored for long. Considering the range of its benefits, it was obvious that all these effects must be explained through the action of various active components of *C. sativa* not on a single target in the brain but rather on a more complex entity.

The ECS has been placed in the anti-cancer spotlight in the last decade. The immense data load published on its dual role in both tumorigenesis and inhibition of tumor growth and metastatic spread has made it into attractive new target for the treatment of various cancer subtypes. Although the search for cancer biomarkers usually favors single targets that enable the exploitation of a biochemical or genetic weakness, marking the vast ECS as a pharmacologically targetable entity brings as many advantages as complications. This review will explore the potential of the ECS in the anti-cancer setting as well as the clinical and ethical pitfalls behind it, and will develop on the value of cannabinoid receptor heteromers as potential new targets for anti-cancer therapies and as prognostic biomarkers.

## The Discovery of the Cannabinoid Receptors

The first components of the ECS that were discovered were THC target sites in the brain. These receptors were named cannabinoid receptors (CBR), but it was still unknown that they represent one of the most abundant neurotransmitter receptors in the whole organism. The first discovered and cloned receptor was named CB1R (Matsuda et al., 1990) and it was concluded that THC exerts a presynaptic inhibition of normal endogenous signaling of this receptor leading to the known psychotropic effects in the central nervous system (CNS) (Roth, 1978). The second identified ECS receptor was found in non-CNS sites, mostly on cells of the immune system, and was named CB2R (Munro et al., 1993). This discovery was a promising start for the search of the other factors involved in the non-psychotropic effects of cannabinoids, as it was found that ligands of CB2R are responsible for their observed immunomodulatory effects (Acharya et al., 2017). Both receptors are expressed in the periphery as well, regulating vital processes in the gastrointestinum, lungs, skin, kidneys, reproductive system, liver, lymph nodes, bones, etc.

The human central cannabinoid receptor gene 1 (CNR1) is located on chromosome 6 (6q15, HGNC ID: 2159) (HUGO), and it encodes three isoforms of a 60 kDa CB1R protein (UniProtKB – P21554) (UniProtKB). It is expressed in CNS areas that control motor behavior, memory and learning, emotions, sensory and endocrine functions, as well as in peripheral nerves and in other extra-neural locations (Atwood and Mackie, 2010). CB1R are mostly located presynaptically and mainly coupled to inhibitory Gi/o proteins, which among other effects inhibit adenylate cyclase and lead to a decrease of intracellular cAMP levels. The activation of CB1R at glutaminergic and GABAergic neuron membranes by endocannabinoids inhibits the release of excitatory and inhibitory neurotransmitters (glutamate, GABA, acetylcholine, dopamine, D-aspartate, noradrenaline, etc.). Thus, CB1R and its ligands contribute to the plasticity of neurotransmission, and are attractive pharmacological targets.

The human central cannabinoid receptor gene 2 (CNR2) is located on chromosome 1 (1p36.11, HGNC ID: 2160) (HUGOb) and it encodes multiple isoforms of a 40 kDa CB2R protein (UniProtKB – P34972) (UniProtKBb). CB2R are also mostly located presynaptically and mainly coupled to inhibitory Gi/o proteins, so 2-AG and other CB2R ligands have various roles in the regulation of immune responses and peripheral neurotransmission. The CB2R was firstly detected in the immune system (Pertwee et al., 2010), but over time its expression was also detected in other cell types. The presence of CB2R in microglial cells during neuroinflammation has been well documented, but it has also been detected in astrocytes and some subpopulations of neurons (Munro et al., 1993; Fernandez-Ruiz et al., 2007; Atwood and Mackie, 2010). As agonists that bind to CB2R usually lack the psychotropic effects seen upon CB1R agonist-based treatments, the selective targeting of CB2R in the CNS and the periphery might be a perspective approach for the treatment of various neurological disorders.

### CB1R and CB2R Are GPCRs

Cannabinoid receptors belong to an extensive family of class A rhodopsin-like G-protein coupled receptors (GPCR), important for transducing signals from the extracellular space to a variety of intracellular signaling molecules (Matsuda et al., 1990; Munro et al., 1993; Pertwee et al., 2010). It is not by chance that the ECS receptors are GPCRs, as the chemical variety of ligands that bind and activate them is extraordinary. GPCRs form the most extensive human membrane protein family, with about 826 members in the human proteome (Cvicek et al., 2016; Shimada et al., 2018). According to their sequence homology, phylogenetic analyses and after the Human Genome Project was completed, Fredriksson et al. (2003) classified GPCRs into five families: rhodopsin (class A, 701 members), secretin (class B, 15 members), glutamate (class C, 15 members), adhesion (24 members) and frizzled/taste (class F, 24 members). Currently, IUPHAR identifies six main classes: the A–F classes, containing human receptors, and classes D and E, including GPCRs of other species with no human orthologs (Sharman and Mpamhanga, 2011). GPCRs are main players in cell communication and transduce sensory signals of external origin, such as odorants and taste molecules, light and endogenous stimuli such as hormones, neuropeptides, neurotransmitters, purine ligands, chemokines, calcium ions, etc. Recently, conformational modifications that mediate GPCR activation have been described, as well as the structural conformation of the receptors necessary to interact with the three classes of proteins that preferentially bind to activated GPCRs: heterotrimeric G proteins, GPCR kinases and β-arrestins (Vilardaga et al., 2010; González et al., 2014; Wells, 2014; Gurevich and Gurevich, 2017; Wang et al., 2017).

G protein-coupled receptors, also known as heptahelical or seven-transmembrane (TM) receptors, are cell surface proteins with a common topology comprised by an extracellular (EC) N-terminus and a cytoplasmic C-terminus with an amphiphilic α-helix (H8) alongside the cell membrane. They have 7 TM α-helices connected by 3 intracellular (ICL) and 3 extracellular loops (ECL) and a disulphide bridge between ECL2 and TM3 (González et al., 2014; Baltoumas et al., 2016). The 7TM bundle can be divided into EC and IC regions. The N-terminus varies in length from relatively short and unstructured (such as many class A members) to long and with many globular domains and conserved secondary structure (such as class C members). The IC module includes the ICLs, a short helix 8 and an IC C-terminus (Katritch et al., 2012; Di Pizio et al., 2016); it is more conserved among GPCRs, in contrast to the EC region, and is responsible for interaction with intracellular proteins implicated in feedback modulation and signal transduction of receptor function (Moreira, 2014; Di Pizio et al., 2016).

The orthosteric binding site of a receptor is the canonical site where the natural ligand binds. Its location can vary markedly between different subclasses of GPCRs and can be found in different positions within the TM bundle or in an extracellular pocket. Ligand binding to this orthorteric site produces conformational changes in the receptor’s cytosolic region facilitating binding and the activation of downstream signaling effectors such as β-arrestins and G proteins (Staus et al., 2016). The orthosteric binding pocket can bind both native or synthetic ligands termed orthosteric ligands. Other ligands targeting GPCRs are currently classified as allosteric, bitopic/dualsteric or bivalent ligands (Feng et al., 2015).

However, elucidation of the GPCRs crystal structures, along with molecular modeling and functional studies, provides insight into the binding mode of natural or synthetic ligands and open new opportunities for the design of next-generation GPCR drugs. In the last years, the number of receptors that have been crystallized has greatly increased and more than 250 structures of 50 different GPCRs have been solved in complex with agonists, antagonists, antibodies, peptides or G proteins (Venkatakrishnan et al., 2013; Shonberg et al., 2015; Shimada et al., 2018). From the elucidated crystal structures of class A GPCRs, it is deduced that ligand binding occurs in a main pocket found between the EC segments of TMs 3, 5, 6, and 7 or in a minor cavity found between the EC segments of TMs 1, 2, 3, and 7 (Gonzalez et al., 2014). Although GPCRs present these common cavities, different ligands can penetrate to various depths within the TM bundle, so that this structural core binds ligands in the EC module and transfers the information to the IC module. It is the most conserved structural component of GPCRs, showing characteristic hydrophobic patterns and functional motifs (Katritch et al., 2012; Di Pizio et al., 2016). Structural studies with class A receptors, have revealed the existence of a conformational change upon receptor activation which include a breaking of “the ionic lock formed between TM6 and the D(E)RY motif in TM3, and movements of the TM5 and TM6 cytoplasmic segments and the ICL3,” with some minor rearrangements in the TM3 helix (Baltoumas et al., 2016).

In the case of cannabinoid receptors, unbiased molecular dynamics has demonstrated that a ligand can penetrate the binding pocket of a class A GPCR via the lipid bilayer. Effectively, Hurst et al. (2010), using microsecond time scale all-atom molecular dynamics (MD) simulations, showed that the endogenous cannabinoid *sn*-2-arachidonoylglycerol (2-AG) accesses to the CB2R passing throughout the lipid bilayer. Their results suggest that 2-AG penetrates the CB2R binding pocket by passing between TM6 and TM7 (Hurst et al., 2010). In 2016, Shao et al. (2016) crystallized the human CB1R thermostabilized by the inverse agonist taranabant, and solved its atomic structure at 2.6-Å resolution. They confirmed a gap between TM1 and TM7 in the EC leaflet that could contribute to a membrane-embedded access channel for lipophilic agonists of CBRs. In order to facilitate the entry of ligands, a subsequent dilatation of the conserved residues (Ile119^1.35^, Phe381^7.37^, and Met384^7.40^) would be required. At the same time, Hua et al. (2016) crystallized CB1R bound to AM6538, an antagonist with a nitrate group substituted on the chlorophenyl moiety of rimonabant. The two structures are in good agreement, but the taranabant-bound and AM6538-bound CB1R structures represent an inactive conformation with respect to G-protein binding. Later, Hua et al. (2017) reported two crystal structures of human CB1R in complex with agonists: a hexahydrocannabinol (AM841) and a tetrahydrocannabinol (AM11542), with a resolution of 2.95 and 2.80 Å, respectively. These CB1R-agonist complexes showed relevant conformational changes compared with the CB1R-antagonist structures, that is, a 53% reduction in the volume of the ligand-binding pocket and an increment in the surface of the G-protein binding region (Hua et al., 2017).

### GPCRs Form Oligomeric Protein Complexes

Since the early 80s the concept on intramembrane receptor-receptor interactions was introduced and the first experimental evidence was reported for their existence in crude membrane preparations from different CNS areas (Agnati et al., 1980; Fuxe et al., 1981; Fuxe and Agnati, 1985). Since then, many evidence show that GPCRs do not act exclusively as monomeric proteins (Agnati et al., 2005; Fuxe et al., 2007). Kniazeff et al. (2011) reviewed that dimer formation is a prerequisite for canonical receptor function in class C GPCRs, and Xue et al. (2015) demonstrated that the mGluR2 dimer interface switches from TM4-TM5 in the inactive state to TM6-TM6 interactions in the active conformation, revealing a key step in class C GPCR activation. In the large subfamily A of GPCRs, although several receptors are able to operate as monomers (Chabre and le Maire, 2005; Ernst et al., 2007; Hanson et al., 2007; Whorton et al., 2007, 2008; Kuszak et al., 2009; Arcemisbéhère et al., 2010; Bayburt et al., 2011), experimental data have shown that most receptors could be expressed as a mixture of monomers and homodimers/oligomers (Teichmann et al., 2014; Vischer et al., 2015; Franco et al., 2016; Nemoto et al., 2016), and that oligomerization is necessary for the maturation of the receptor, its inclusion in the membrane and its function (Angers et al., 2000, 2002; Fotiadis et al., 2003; Herrick-Davis et al., 2006; Lopez-Gimenez et al., 2007; Han et al., 2009; Wade et al., 2011; Ng et al., 2013; Hurevich et al., 2014; Liste et al., 2015; Gahbauer and Böckmann, 2016; Lao et al., 2017; Jin et al., 2018). Fung et al. (2009) reported receptor oligomers, mostly with tetrameric structure, of β2-adrenergic receptors after reconstitution into phospholipid vesicles. Albizu et al. (2010) demonstrated the *in vivo* existence of native oxytocin receptor dimers and Herrick-Davis et al. (2012, 2013, 2015) provided strong experimental evidence that adrenergic, muscarinic, dopamine, and serotonin 5-HT_2C_ receptors form homodimers endogenously expressed in their cellular environment. Using dual color photoactivation localization microscopy with photoactivatable dyes, Jonas et al. (2015) reported that the luteinizing hormone receptor (LHR) is organized in a mixture of monomers, dimers and lower order oligomers at the plasma membrane with distinct spatial geometries. Likewise, M1 muscarinic receptor, D2 dopamine receptor, β_1_-adrenergic receptor and chemokine receptor (CXCR4) can exist in a dynamic equilibrium between monomers/dimers/oligomers, that can be regulated by selective agonists, antagonists or bivalent ligands (Calebiro et al., 2013; Pediani et al., 2016; Tabor et al., 2016). Cai et al. (2017) demonstrated monomer-to-dimer interconversion of the class A apelin receptor (APJ) on the cell membrane, with APJ dimers possessing new functional characteristics after agonist activation, such as distinct G-protein binding profile and cell responses. Recently, Parmar et al. (2017), using BRET, bimolecular fluorescence complementation (BiFC) and fluorescence correlation spectroscopy (FCS) indicated that the β_2_-adenergic receptors are predominantly homodimers, and Jastrzebska et al. (2017) demonstrated that the human red cone opsin forms a stable dimer in the live cell membrane with the existence of three essential amino acids, I230, A233 and M236 that are required for dimerization.

Furthermore, a multitude of other class A GPCRs have been also found to form homomers, specially in the last decade, such as cannabinoid CB_1_, adenosine A_1_, A_2A_ and A_3_, adrenergic α_1B_, dopamine D_1_ and D_3_, serotonin 5HT_1A_, 5HT_2A_, and 5HT_7_, δ, κ and µ opioid, angiotensin AT_1_, muscarinic M_2_ and M_3_, melatonin MT_2_, and niacin receptors (Cvejic and Devi, 1997; Jordan and Devi, 1999; He et al., 2002; Ayoub et al., 2004; Goin and Nathanson, 2006; Łukasiewicz et al., 2007; Mandrika et al., 2010; Teitler et al., 2010; Gracia et al., 2011, 2013; May et al., 2011; Pou et al., 2012; Szalai et al., 2012; Herrick-Davis et al., 2013; Guitart et al., 2014; Bonaventura et al., 2015; Bagher et al., 2017).

G protein-coupled receptors are not only present as monomers and homomers but also form heteromers with other GPCRs (Smith and Milligan, 2010; Ferré et al., 2014; Gomes et al., 2016a; Farran, 2017; Gaitonde and González-Maeso, 2017; Guidolin et al., 2018). GPCR heteromers with two or more receptor protomers are macromolecular complexes with biochemical properties clearly different from those of its individual components (Ferré et al., 2009, 2014; Gomes et al., 2016a). Three consensus criteria have been published by the International Union of Basic and Clinical Pharmacology to classify a true GPCR heteromers (Kenakin et al., 2010). The first criterion indicates that the heteromer components must co-localize to the same subcellular compartment and physically interact in native tissues (Albizu et al., 2010; Hounsou et al., 2015; Gomes et al., 2016a). The second consensus criteria requires that the heteromers must exhibit specific properties, that differ from those associated with the individual protomers, such as trafficking, ligand binding and signaling (biochemical fingerprint) (Jordan and Devi, 1999; Hillion et al., 2002; Terrillon et al., 2004; Sohy et al., 2007; Gomes et al., 2011, 2016a; González et al., 2012; Kern et al., 2012; Bellot et al., 2015; Jonas et al., 2018). Finally, criterion 3 postulates that heteromer disruption brings about a loss of interaction and, therefore, to a loss of the typical biochemical fingerprint of the heteromer (Baba et al., 2013; Fujita et al., 2015; Gomes et al., 2016a). Respect to the second criterion, according to Cortés et al. (2016), there are three major allosteric modulations in GPCR heteromers that imply alterations of the affinity and/or efficacy of a ligand by a protomer within the heteromer due to binding of an allosteric modulator in any site of the heteromer, or to binding of an orthosteric ligand in the other protomer or to the simple presence of the other protomer within the heteromer (Kenakin and Miller, 2010; Wootten et al., 2013; Ferré et al., 2014; Ferré, 2015; Casadó-Anguera et al., 2016).

Although many GPCR heteromers have been identified using heterologous cell lines, only very few fit all three criteria. This is mainly due to the difficulties to study these structures in native tissues because of the lack of sensitive and selective enough tools, able to detect *in vivo* evidence of these endogenous heteromers and to demonstrate that they are close enough to interact (Gomes et al., 2016b). Therefore, the accomplishment of at least two out of three criteria is required for the acceptance of a GPCR heteromer (Jonas and Hanyaloglu, 2017). The main strategies to target GPCR heteromers are the generation of selective compounds, which exhibit higher efficacy in tissues from wild type animals or in cells expressing both receptors than in knock-out animals tissues or in cell only expressing the individual receptors (Akgün et al., 2013; Gomes et al., 2013a; Molero et al., 2015; Nimczick and Decker, 2015; Peterson et al., 2017). Likewise, the use of membrane-permeable peptides that target the dimerization interface or heteromer-selective antibodies that can recognize an epitope in the heteromer but not in the individual protomers, have been most useful to detect GPCRs heteromers *in vivo* (Gupta et al., 2010; He et al., 2011; Viñals et al., 2015; Gomes et al., 2016a; Moreno et al., 2017; Jacobs et al., 2018). Finally, it is important to note the existence of single-molecule techniques, which are ideal for understanding the conformational complexity and dynamic signaling of GPCRs (Tian et al., 2017). Their application in living cells can provide the tools to directly visualize individual receptors in homomers and heteromers and how GPCRs can move about and interact in the presence of diverse ligands (Jonas et al., 2016; Scarselli et al., 2016; Calebiro and Sungkaworn, 2018).

A basic heterotetrameric structure formed by two homomers of a G_s_-coupled and a G_i_-coupled receptor has been reported for the dopamine D1-D3 receptor heteromer (Guitart et al., 2014), the adenosine A1-A2A receptor heteromer (Navarro et al., 2016), the adenosine A2A-dopamine D2 receptor heteromer (Bonaventura et al., 2015; Casadó-Anguera et al., 2016; Borroto-Escuela et al., 2018; Navarro et al., 2018), and the adenosine A1-dopamine D1 receptor heteromer (Rivera-Oliver et al., 2018).

#### Cannabinoid Receptors Heteromers

Cannabinoid receptors have been described for a long time as constituents of particular GPCR receptor heteromers. CB1Rs have been demonstrated in the past to interact with other GPCRs, such as adenosine A_2A_ receptors (Carriba et al., 2007; Moreno et al., 2018), dopamine D_2_ receptors (producing a change in the coupling from Gi to Gs) (Glass and Felder, 1997; Kearn et al., 2005; Marcellino et al., 2008), D_2_ and adenosine A2A receptors at the same time (generating a negative modulation of the function of D_2_ receptor by A2A and CB1 agonists) (Carriba et al., 2008; Navarro et al., 2010; Bonaventura et al., 2014), opioid µ and δ receptors (producing a negative cross-talk between receptors) (Rios et al., 2006), orexin OX_1_ receptors (producing a positive cross-talk and cross-antagonism by orexin) (Hilairet et al., 2003; Ellis et al., 2006), angiotensin AT_1_ receptors (with an increase of AT_1_ receptor signaling) (Rozenfeld et al., 2011), CB2R (negative cross-talk and bidirectional cross-antagonism in neuronal cells in culture and *in vivo* is produced by the coactivation of both receptors) (Callén et al., 2012), adrenergic β_2_ receptor (adrenergic agonists inducing CB1R internalization) (Hudson et al., 2010), and 5HT_2A_ serotonin receptor (Viñals et al., 2015). Also, CB1R can form heteromers with the cannabinoid-related orphan receptor GPR55 in HEK-293 cells (Kargl et al., 2012; Martínez-Pinilla et al., 2014). Contrary to CB1R, not much is known about the existence and functional importance of heteromers concerning CB2R, however, there is evidence of its interaction with GPR55 in transfected cells (Balenga et al., 2014) and with CXCR4 (Coke et al., 2016; Scarlett et al., 2018).

## The Discovery of Endocannabinoids

The first discovered and most abundant endocannabinoids were anandamide (N-arachidonoyl ethanolamide, AEA) (Devane et al., 1992) and 2-arachydonoyl glycerol (2-AG) (Mechoulam et al., 1995), derivatives of ω-6 arachidonic acid (ARA), an essential polyunsaturated fatty acid (PUFA). Beside AEA and 2-AG, other unsaturated lipid-based molecules have been classified as endocannabinoids due to their function and comparably high levels (N-arachidonoyl dopamine, N-oleoyldopamine, homo linoleoyl ethanolamide (HEA), docosa tetraenyl ethanolamide, virodhamine, noladin ether, palmitoyl ethanolamide, oleoylethanolamide, oleamide, eicosapentaenoylethanolamide, sphingosine, hemopressin, etc.) (Pertwee, 2015; Nikan et al., 2016). The significance of these small molecules is highlighted by the fact that their signaling pathways exist even in very primitive organisms where they regulate a range of vital processes (De Petrocellis et al., 1999). They are produced and are active inside the body upon demand, and they exert a regulatory role of adaptive cellular responses to various endogenous and environmental stimuli that endanger internal homeostasis.

Endocannabinoid signaling is not a classical example of neurotransmission, as their effects are mostly restricted to local sites of their biosynthesis and release. Their biosynthesis precedes the stimuli, and they are stored in synaptic vesicles until needed. Once they have exerted their effect on the receptors of postsynaptic neurons, they are transported back to the presynaptic neuron terminating the short-lived response to a stimulus (Lu and Mackie, 2016). As exogenous cannabinoids are usually supplied in excess they can take over the endocannabinoid signaling for longer time periods, leading to a range of physiological effects. Beside CB1R and CB2R, other receptors have been found to be the targets of endocannabinoids and exocannabinoids in the CNS and in tumor tissues, including the transient receptor potential channels, ligand and voltage-gated ion channels and other orphan G protein-coupled receptors as GPR55, GPR18 and GPR119 (Soderstrom et al., 2017). Receptor-independent regulatory effects of endocannabinoids have also been documented, contributing to the plasticity of the ECS (Lu and Mackie, 2016).

### Non-ARA Based Endocannabinoids

Beside the most common endocannabinoid derivatives of ARA, other classes of lipid-based molecules important for the ECS have been detected in high enough levels to be classified as endocannabinoids (McDougle et al., 2017). ω-3 PUFAs, such as docosahexaenoic acid (DHA) and eicosapentaenoic acid (EPA), convert to a range of biologically active molecules important for proper functioning of essential biochemical processes in the body. These epoxyeicosatetraenoic acid-ethanolamides (EEQ-EA) and epoxydocosapentaenoic acid-ethanolamides (EDP-EA) are formed through the action of cytochrome P450 epoxygenase, and have been found to preferentially stimulate CB2Rs and induce anti-inflammatory and antiangiogenic effects (McDougle et al., 2017). The complexity of the ECS can also be seen in the constant interplay of ω-3 and ω-6 PUFA based endocannabinoids, as well as in the sharing of the biosynthetic/degradative and regulatory levels by various classes of endocannabinoids, which influences the final outcome of a specific stimuli (Dyall, 2017).

### Binding of Endo- and Exocannabinoids to Cannabinoid Receptors

Exogenous cannabinoids (plant-derived and synthetic) bind to CBRs as their binding pocket is flexible and can interact with ligands that are not the exact size and shape match to their designated endocannabinoids (Sim-Selley, 2003). As a consequence, all the processes regulated by endocannabinoids are susceptible to the interference of exogenous cannabinoids (allosteric, bitopic/dualsteric or bivalent ligands), and their effects have been extensively documented in various settings (pregnancy/infertility, motor functions, CNS development, etc). THC binds to CB1R instead of anandamide on neurons, but also to CB2R on immune cells which changes the endogenous response to infection and also reduces inflammation. 2-AG also has a mimetic plant cannabinoid called cannabidiol (CBD) which exerts its mostly health promoting effects by competing with 2-AG for its binding places on CB2R and other receptors (Iffland and Grotenhermen, 2017). These effects are even more pronounced in conditions where ECS signaling is impaired (Clinical Endocannabinoid Deficiency) due to external factors as stress, diet, medicaments or internal ones involving a dysbalance between the biosynthesis, breakdown and transport of endocannabinoids (Russo, 2016). Thus, the observed clinical benefits of exogenous cannabinoids have a strong biochemical rational.

However, the effects of exocannabinoids cannot be considered as a simple receptor-takeover as they do not act as mere substitutes for endocannabinoids. Depending on their concentration, abundance of receptors and the levels and activity of endogenous ligands and enzymes, agonism, antagonism and inverse agonism of cannabinoid and non-cannabinoid receptors can occur (Pertwee, 2015; Lu and Mackie, 2016). *In vitro* evidence shows that around 13 endogenous compounds have been detected so far that can bind to these receptors orthosterically or allosterically (Pertwee, 2015), while AEA and 2-AG are the main orthosteric ligands.

Apart from orthosteric ligands, several allosteric modulators for CBRs have been discovered. While cannabinoids interact with the orthosteric receptor site, these novel CBR ligands bind the receptor at sites topographically distinct from the orthosteric binding site, modifying the receptor conformation and leading its novel properties and modes of action (Conn et al., 2009; Fay and Farrens, 2013; Shore et al., 2014; Stornaiuolo et al., 2015; Foster and Conn, 2017). The allosteric cannabinoid ligands modulate the CBR activity by altering the affinity and/or the efficacy of an orthosteric ligand in either a positive (positive allosteric modulator, PAM) or negative (negative allosteric modulator, NAM) manner. In the last decade, many allosteric modulators for CB1R have been reported (Nguyen et al., 2017; Scott and Kendall, 2017; Dopart et al., 2018). Examples of CB1R NAM are ORG27569, ORG27759, and ORG29647 (Price et al., 2005; Ahn et al., 2013), PSNCBAM-1 (Horswill et al., 2007), pepcans (Bauer et al., 2012), pregnenolone (Vallée et al., 2014) and CBD (Laprairie et al., 2015). CB1R PAM are RTI-371 (Navarro et al., 2009), lipoxin A4 (Pamplona et al., 2012), GAT211 and its enantiomer GAT229 (Laprairie et al., 2017b), and ZCZ011 (Ignatowska-Jankowska et al., 2015). Recently, some modulators of CB2R have also been described; Martínez-Pinilla et al. (2017) reported that CBD could also act as a NAM of CB2R because it is able to negatively modulate the binding and functional effect of CB2R agonists; Petrucci et al. (2017) reported that pepcan-12 is a potent CB2R PAM and, very recently, Pandey et al. (2019) described DHGA and TBC as CB2R NAMs, decreasing the binding of the orthosteric agonist CP55,940. The therapeutic usefulness of these cannabinoid allosteric modulators is emerging, and they offer an exciting potential for mechanistic analyses and for the development of therapeutics (Scott and Kendall, 2017).

## The Discovery of the Endocannabinoid Enzymatic and Transport Systems

The last ECS component that was described in more detail was the enzymatic machinery behind the biosynthesis, degradation and transport of endocannabinoids. Endocannabinoids are lipid neuromodulators produced on-demand making the ECS a fast acting and adaptive entity. Anandamide and 2-arachydonoyl glycerol have very similar chemical structures (Figure 1) but the pathways involved in their biosynthesis and degradation are completely different, highlighting their distinct physiological roles. They are both derivatives of arachidonic acid, an essential PUFA, but while anandamide is synthesized mostly from N-arachidonoyl phosphatidyl ethanol by various pathways, 2-AG is produced mainly from phosphatidyl inositol bis-phosphate in a calcium-dependent manner involving phospholipase C (PLC) and diacylglycerol lipase (DAGL) (Lu and Mackie, 2016). Also, fatty acid amide hydrolase (FAAH) primarily hydrolyzes AEA in the endoplasmic reticulum, and monoacylglycerol lipase (MAGL) is the primary hydrolytic degrader of 2-AG, with FAAH and some serine hydrolases (ABHD6 and ABHD12) as proposed secondary degraders (Savinainen et al., 2012). Levels of endogenous cannabinoids depend also on the activity of their uptake and effective transport in the cell, as they are hydrophobic and cannot diffuse through the cytosol and cell membrane easily. Many carriers and molecules have been implicated in this process depending on the cell type, as heat shock proteins, serum albumin and other fatty-acid binding proteins as FAAH-like anandamide transporter (FLAT), cholesterol, ceramides (Nicolussi and Gertsch, 2015; Di Scala et al., 2018). Once AEA reaches the target receptors on cells, and exerts its effects, it is recycled back to the cytoplasm and is subjected to enzymatic hydrolysis. Some exogenous cannabinoids, as CBD for example, can interfere with AEA FAAH-mediated breakdown, raising the levels of available AEA, thus indirectly inducing various non-psychoactive effects (Bisogno et al., 2001).

## ECS Components in Anti-Cancer Therapy

The mechanisms involved in the regulation of ECS as well as the processes that it regulates include practically every pathway important in cancer biology. So it is not a matter of chance that ECS components can exert antiproliferative, proapoptotic, antiangiogenic, anti-metastatic and anti-inflammatory effects depending on tumor type and specific setting. Cannabinoid receptors and endocannabinoids are generally up-regulated in tumors (Sanchez et al., 2001; Guzmán, 2003; Caffarel et al., 2006; Malfitano et al., 2011), and their expression levels can be linked to tumor aggressiveness (Nomura et al., 2010; Thors et al., 2010; Malfitano et al., 2011). These data imply that an over-activation of ECS might be a pro-tumorigenic factor (Malfitano et al., 2011), but considering the complexity of this system, the effects it induces depend on many factors. The various implications of the ECS in different cancer types have been reviewed in Table 1.

**Table 1:** The implication of the endocannabinoid system in different cancer types.

ECS component	Type of effect	Cancer type	References
CB1R	Gene/Protein overexpression	Glioma, astrocytoma	Galve-Roperh et al., 2000; Cudaback et al., 2010
CB2R	Gene/Protein overexpression	Glioma, melanoma, astrocytoma, breast, hepatic, pancreatic cancer	Sanchez et al., 2001; Lorente et al., 2011; Velasco et al., 2012
CB2R-CXCR4	Heteromerization	Breast, prostate cancer	Coke et al., 2016; Scarlett et al., 2018
CB2R-GPR55	Heteromerization	Breast cancer	Moreno et al., 2014
HER2-CB2R	Heteromerization	Breast cancer	Pérez-Gómez et al., 2015; Blasco-Benito et al., 2019
Anandamide	Upregulation	Colon cancer	Patsos et al., 2010
FAAH	Gene/Protein overexpression	Prostate cancer	Thors et al., 2010
MAGL	Gene/Protein overexpression	Breast, ovarian, melanoma, colorectal	Granchi et al., 2017; Pagano et al., 2017


### Cannabinoid Receptors in Cancer

There are *in vivo* reports showing that genetic ablation of CB1R and CB2R leads lower the skin-cancer inducing potential of UV light (Zheng et al., 2008), and that over-expression of CB2R contributes to a higher risk of leukemia upon leukemia virus infection (Joosten et al., 2002). On the contrary, there are reports implying that pharmacological activation of CBRs leads to a reduction of tumor growth (Guzmán, 2003; Sarfaraz et al., 2008), suggesting that ECS signaling might induce tumor-suppressive effects. This opinion is reinforced by *in vivo* reports that the deletion of CB1R accelerates tumor growth (Wang et al., 2008), that the presence of higher endocannabinoid levels lead to a reduction of precancerous lesions (Izzo et al., 2008), and that lower expression of MAGL decreases tumor growth (Nomura et al., 2010; Velasco et al., 2015).

The expression of CB1R and CB2R in cancer cells and the cells originating from the same tissue often does not correlate well (Guzmán et al., 2006; Fernandez-Ruiz et al., 2007; Sarfaraz et al., 2008; Velasco et al., 2012). Different types of tumor cells can over-express CB1R and/or CB2R and are as such very attractive for various anti-cancer approaches (Pagano and Borrelli, 2017). On the other side, there are reports that the loss of expression of CB1R and/or CB2R can lead to acceleration of tumor growth (Wang et al., 2008). Thus, their significance as prognostic and/or predictive factors for targeted therapies must be explored in more detail for each cancer subtype, especially when biased agonism is taken into account (Laprairie et al., 2017a). Exploring the ECS non-receptor mechanisms is also a promising approach, as receptor expression does not have a straight forward correlation with tumorigenicity (Soderstrom et al., 2017). There are reports that the activation of CBRs leads to apoptosis of tumor cells and inhibition of their dissemination through the regulation of RAS/MAPK and PI3K–AKT pathways (Pagano and Borrelli, 2017), TNFα-induced ceramide synthesis (Cianchi et al., 2008), endoplasmic reticulum stress-related genes (Carracedo et al., 2006a), COX-2-dependent cell death (Patsos et al., 2010), inhibition of neo-angiogenesis (Pisanti et al., 2007), and many other proposed mechanisms.

### Heteromers as Potential Targets

Because GPCRs allosterically facilitate the transfer of information across the cytoplasmic membrane responding to extracellular signals, they acquire an essential role in the mediation of signal transduction. In turn, this renders GPCRs therapeutic targets in a wide number of diseases, either due to their capacity to regulate a set of signaling cascades implicated in a specific disease or due to their direct involvement in the pathophysiology of this disease (Lefkowitz, 2004; Cvicek et al., 2016). Over 90% of described GPCRs are expressed in the CNS and are crucial for the appropriate functioning of many neurological actions (Vassilatis et al., 2003). Thus, it is not surprising that, in clinical medicine, they are the most important class of membrane proteins. Currently, more than 30% of all US Food and Drug Administration (FDA) approved drugs target GPCRs (Shimada et al., 2018), and these drugs are utilized in a wide range of therapeutic areas, including cancer, inflammation and diseases of the CNS, cardiovascular, respiratory and gastrointestinal dysfunctions, diabetes, obesity, and pain (Feng et al., 2015). Hauser et al. (2017) pointed out that more than 300 agents were in clinical trials in that moment, of which around 60 targeted novel GPCRs for which no drug had yet been approved.

Nevertheless, despite “the proven success of GPCRs as drug targets, clinically useful ligands do not exist for the majority of GPCRs” (Ferré et al., 2014). This is probably because the true target of the GPCRs are not the individual (monomeric) receptors but heteromeric complexes with other GPCRs, other receptors or other membrane, cytosolic or extracellular proteins, in general. Thus, the monomeric strategy is no longer the most appropriate for developing clinically useful drugs and we hypothesize that the approach should be focused on heteromeric GPCRs. “A particular advantage of receptor heteromers as targets for drug development is that they can be involved in the pathophysiology of the disorder being targeted. Thus, the receptor heteromer would be more likely to be disease-specific than would the corresponding monomeric/homomeric receptors” (Cortés et al., 2016). Newer reports show that heteromer-selective drugs can exist in the form of small-molecules, bivalent or multifunctional ligands, or antibodies, displaying higher affinity and efficacy for a receptor that forms a certain heteromer than for this receptor in another heteromer or in the monomeric form (Orru et al., 2011; Gomes et al., 2013c,b, 2016a; Cortés et al., 2016; Pulido et al., 2018; Qian et al., 2018a,b).

In addition to heteromerization, drug discovery efforts for cannabinoid receptors also involve the use of allosteric cannabinoid ligands. One of the main problems of the use of orthosteric ligands is their lack of selectivity among the different members of a GPCR subfamily, because the orthosteric binding sites are highly conserved (Conn et al., 2009). However, the alosteric binding sites are less conserved and show greater selectivity across different receptor subtypes (Conn et al., 2009). The development of allosteric modulators of GPCRs in general, and of CBRs in particular, have emerged as new approaches for developing therapeutic drugs that may be useful for the treatment of CNS disorders, without the inherent side effects of orthosteric ligands (Foster and Conn, 2017; Nguyen et al., 2017; Scott and Kendall, 2017). The development of biased allosteric modulators of CB1R has also been showed as a promising way to selectively modulate a therapeutically desirable CB1R signaling pathway (Khajehali et al., 2015). Furthermore, there is the possibility that the dimerization of GPCRs, and CBRs, could lead to the appearance of an allosteric binding site, specific for the dimer and non-existent in the individual protomers. This new allosteric site has been reported for homocysteine in the A2AR-D2R heterodimer (Agnati et al., 2006; Cervetto et al., 2018).

#### GXCR4-CB2R Heteromers in Cancer

It has been reported that CB2R can form heteromers with the G protein-coupled chemokine receptor CXCR4 in human breast and prostate cancer cells (Coke et al., 2016; Scarlett et al., 2018). CXCR4 is implicated in various mechanism that enhance the cell’s ability to proliferate and migrate, thus its activation has been linked to local and distant metastatic invasion. Upon the *in vitro* application of both CXCR4 and CB2R agonists however, a reduction of the cell’s CXCR4-induced ERK1/2 dependent migration has been documented, most likely due to the presence of functional CXCR4-CB2R heteromers (Coke et al., 2016). This heteromerization might enable cannabinoids to indirectly reduce the invasive properties of cancer cells by inhibiting the effects of CXCR4 agonists. The presence of CXCR4 and CB2R agonists has also been associated with the inhibition of the Gα13/RhoA signaling pathway in prostate cancer cells. The levels of RhoA and Gα13 proteins decreased upon this dual stimulation, and led to an abolishment of the cytoskeletal changes necessary for directional cell migration (Scarlett et al., 2018). The invasive potential of the cells was further decreased by an observed reduction of integrin α5 expression *in vitro*, which is crucial for the cell’s ability to adhere to the extracellular matrix. Over all, all these data identify a novel pharmacologic target for the modulation of tumor cell migration and invasion in the context of metastatic disease.

#### GPR55-CB2R Heteromers in Cancer

It has also been reported that CB2R and GPR55 are overexpressed in many of cancer types (Henstridge et al., 2011; Velasco et al., 2012), where they are crucial for the regulation of the cell fate (Henstridge et al., 2011; Andradas et al., 2011; Piñeiro et al., 2011; Velasco et al., 2012; Pérez-Gómez et al., 2013). As cannabinoids modulate the activity of GPR55, its anti-cancer effects have been analyzed as well.

CB2R/GPR55 transfected cells and breast cancer cells form GPR55-CB2R heteromers with unique pharmacological and signaling properties (Moreno et al., 2014). The expression of the GPR55-CB2R heteromers influences cannabinoid signaling in a way that their direct targeting using appropriate amounts of THC might lead to a reduction of tumor growth, both *in vitro* and *in vivo*. This is a promising new approach for the development of drugs that target these heteromers in future cancer-related studies. These results also help to explain the potential molecular mechanisms behind the documented but still poorly comprehended biphasic effects of cannabinoids, present in older reports concerning food intake, motor behavior, anxiety, and others (Sulcova et al., 1998; Sañudo-Peña et al., 2000; Moreira and Wotjak, 2010).

It might even be speculated that GPR55-CB2R heteromers could exist and be crucial at other cancer-related sites, such as bones or hematopoietic cells, where their overexpression has been detected (Whyte et al., 2009; Balenga et al., 2011).

#### HER2-CB2R Heteromers in Cancer

In breast cancer, the overexpression of the tyrosine kinase receptor (TKR) human V-Erb-B2 Avian Erythroblastic Leukemia Viral Oncogene Homolog 2 (HER2) is a particular hallmark of one type of this disease (Perou et al., 2000; Sorlie et al., 2001; Sotiriou et al., 2003; Curtis et al., 2012). The activation of TKRs engages crucial signaling pathways implicated in cellular proliferation, development, differentiation, angiogenesis, and other processes (Higgins and Baselga, 2011). Approximately 20–30% of primary breast cancer cells exhibit HER2 gene amplification and protein overexpression which is a poor prognostic biomarker and leads to an inadequate response to chemotherapy (Moasser, 2007). At the same time, CB2R is overexpressed in breast cancer, and it is present in especially high levels in aggressive (high-grade) tumors (Caffarel et al., 2006; Qamri et al., 2009; Caffarel et al., 2010).

There are very few examples of physical interaction between RTKs and GPCRs, although several RTK-RTK heteromers and GPCR-GPCR heteromers have been previously described. Transactivation of RTKs by GPCRs and *vice versa* has been observed, and in most cases physical interactions suggested, but no solid evidence of the existence of heteromers has been demonstrated (Pyne and Pyne, 2011).

The best characterized RTK-GPCR heteromer is produced by HER2 and β2-adrenergic receptors in the heart and it is essential for the cardiac homeostasis (Negro et al., 2006); by fibroblast growth factor receptor and adenosine A2A receptors (Flajolet et al., 2008) or serotonin 5-HT1A receptors (Borroto-Escuela et al., 2012), which take part in synaptic plasticity; and by EGFR and GPR54, which seem to induce breast cancer cell invasiveness (Zajac et al., 2011).

The deregulation of the ECS in many cancers has been extensively documented (Pacher et al., 2006; Alpini and Demorrow, 2009; Pisanti and Bifulco, 2009; Caffarel et al., 2012). Although there is a strong rationale for using CB2R as an anti-cancer drug target (Guindon and Hohmann, 2011; Caffarel et al., 2012; Velasco et al., 2012), details on its impact in tumor development and progression is still lacking. The pro-oncogenic effect of CB2R in HER2+ breast cancer was discovered when HER2-CB2R heteromers were detected in these cells, and having observed a simultaneous appearance of higher CB2 protein expression and poorer overall relapse-free and metastasis-free survival of patients (Pérez-Gómez et al., 2015). When there are no exogenous cannabinoids, CB2R regulates HER2 signaling (Pérez-Gómez et al., 2015), which was an unprecedented finding of CB2R-based control of HER2. Thus, strategies based on the simultaneous targeting of these two receptors (or their shared downstream effectors) may prove effective (Pérez-Gómez et al., 2015). It would potentially also mean that the combination of anti-HER2 drugs and cannabinoids acting on CB2R may induce synergistic anti-cancer effects. Recently, Blasco-Benito et al. (2019) proposed a mechanism controlling the oncogenic activity of HER2 in breast cancer through the HER2-CB2R heteromer. Inactivation and degradation of HER2 and promotion of antitumor responses is produced by the disruption of HER2-CB2R heteromer by THC, which binds selectively to CB2R, or by using a synthetic peptide with the amino acid sequence of specific transmembrane 5 domain (TM5) of the CB2R. All these findings reveal a new mechanism of regulation of HER2 activity, and support the existence HER2-CB2R heteromers as novel therapeutic targets for HER2+ breast cancer (Pérez-Gómez et al., 2015; Blasco-Benito et al., 2019).

**FIGURE 2:**
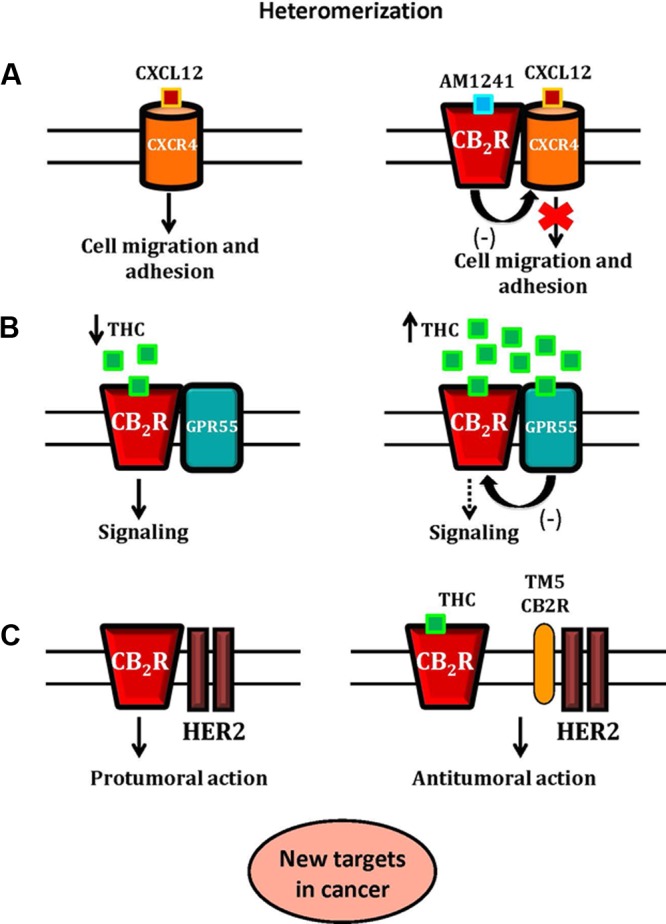
A schematic representation of CB2R-CXCR4, CB2R-GPR55 and CB2R-HER2 heteromers and their role as new targets in cancer. In Panel **(A)**, the activation of CB2R-CXCR4 heteromers inhibits prostate cancer cell migration and adhesion. The binding of CXCL12 to its receptor, CXCR4, induces CXCR4-mediated cell migration and adhesion. The application of both CXCR4 and CB2R agonists inhibits the effect of CXCR4 agonist, due to the presence of functional CB2R-CXCR4 heteromers. In Panel **(B)**, the hypothetical effect of THC on the CB2R-GPR55 heteromer. At low concentrations, THC acts as a CB2R agonist promoting signaling. At higher concentrations, THC targets GPR55, acting as an antagonist, and by cross-antagonism, inhibits CB2R signaling. In Panel **(C)**, the proposed mechanism of control of the CB2R-HER2 heteromer in breast cancer is shown. The HER2-CB2R heteromer is disrupted by THC or by using a synthetic peptide with the amino acid sequence of specific transmembrane 5 domain (TM5) of the CB2R, triggering the inactivation of HER2 and producing antitumor responses.

The protein complexes CXCR4-CB2R, GPR55-CB2R and HER2-CB2R possess particular pharmacological and signaling properties, and their modulation might affect the antitumoral activity of the ECS. So, the cannabinoid receptor heteromers have a promising potential value as new targets for anti-cancer therapies and as prognostic biomarkers (Figure 2).

### Cannabinoid Ligands in Cancer

The use of endocannabinoids as anti-cancer therapeutics has been extensively studied, and it can be concluded that they generally exert protective and beneficiary effects, inhibiting tumor growth and progression and restoring homeostasis. However, endocannabinoids are very important regulatory molecules involved in the metabolism of lipids and general signaling non-related to cancer, and there are reports that their levels can be elevated in some cancer subtypes (Zhang et al., 2016). So they also might also contribute to the pathogenesis and disease progression. Their interaction with standard chemo- and targeted therapies needs to be taken into account when exploring possible concurrent treatments, as they might interfere with the cytotoxic mechanisms in question (Miyato et al., 2009).

Since their discovery in the 1980s and 1990s, a lot of data has been published using various experimental models of cancer (from cell lines to genetically engineered mice) showing that various cannabinoids can have anti-tumor effects (Velasco et al., 2012). Endo- and exocannabinoids, as well as many synthetic cannabinoid receptor agonists have demonstrated this activity. Synthetic cannabinoids can have a comparable affinity for CBRs (WIN 55,212-2, HU-210), or higher affinity for one of them (methanandamide for CB1R, JWH-133 for CB2R). These findings add to the data load documenting that pharmacologic stimulation of CBRs is generally anti-tumorigenic. The induction of cancer cell death by apoptosis and the inhibition of proliferation is usually the mode of action of cannabinoids, in almost every tested cancer cell type (Velasco et al., 2012). Furthermore, it has been shown that cannabinoids reduce tumor angiogenesis as well as invasion/metastasis *in vivo*. However, there are also studies that document tumor-promoting effects of cannabinoids *in vitro* (Hart et al., 2004; Cudaback et al., 2010), and those that suggest that they diminish the immune system’s ability to act as a tumor-suppressor (Zhu et al., 2000; McKallip et al., 2005).

### Cannabinoid Enzymes and Transporters in Cancer

Targeting ECS enzymes and transporters has also been explored as a therapeutic option with limited success, as any change in endocannabinoid biosynthesis, degradation and transport can have unpredictable effects in the organism. Changes in the endocannabinoid metabolic pathways by interfering with enzyme/transporter activity and levels can lead to an increased availability of endocannabinoids and general protective effects, especially in situations where these enzymes are deregulated as a consequence of tumorigenesis, as is the case with MAGL in colorectal cancer (Pagano et al., 2017). Many inhibitors of FAAH, MAGL and transporters are being tested in various experimental and clinical settings, alone and in combination (Ogawa and Kunugi, 2015; Panlilio et al., 2016; Chicca et al., 2017; Granchi et al., 2017), and many more are currently under development. However, a range of non-psychotropic CNS and systemic adverse effects needs to be thoroughly evaluated as learning and memory impairment can occur upon treatment (Panlilio et al., 2016).

## Cannabinoid-Induced Anticancer Mechanisms

### Cancer Cell Death

Antiproliferative action of many plant-derived, endogenous and synthetic cannabinoids has been documented in various *in vitro* cancer models (Bifulco and Di Marzo, 2002; Guzmán, 2003). Also, *in vivo* experiments have confirmed that treatment of nude mice with cannabinoids leads to the formation of various tumor xenografts: lung carcinoma (Munson et al., 1975), thyroid epithelioma (Bifulco et al., 2001), lymphoma (McKallip et al., 2002), skin carcinoma (Casanova et al., 2003) and glioma (Galve-Roperh et al., 2000). Glioma cells have been used as the most common model system for studying cannabinoid-induced anticancer mechanisms. Initial studies showed that cannabinoids can induce apoptosis of glioma cells via CB1R and CB2R dependent *de novo* synthesis of the sphingolipid ceramide which has pro-apoptotic properties (Galve-Roperh et al., 2000; Sanchez et al., 2001; Gomez del Pulgar et al., 2002; Blázquez et al., 2004).

The level of cancer cell death resulting from the activation of CBRs depends on the type of agonist that evoked the response, and rarely on its mere affinity for a given CBR. For example, the synthetic agonist WIN-55,212-2 has a higher affinity for CBRs than THC, but lower concentrations of THC are needed for the comparable cancer cell death-inducing response (Pertwee et al., 2010). Beside the type of agonist, in some cases the type of receptor that is activated determines the resulting outcome. In glioma cells, the same level of inhibition of cannabinoid-induced cell death is achieved by antagonizing CB1R or CB2R, but in pancreatic, breast of hepatic cells only CB2R antagonists are effective (Galve-Roperh et al., 2000; Carracedo et al., 2006a; Caffarel et al., 2006; Lorente et al., 2011; Vara et al., 2011). The mechanisms which regulate these anti-cancer actions depending on agonist and receptor types are still unknown.

Autophagy has proven as one of the crucial effects of CBR agonists by which they promote cancer cell death. There are reports showing that activation of CBRs *in vitro* can lead to autophagy, and inhibition of autophagy via genetic or pharmacological mechanisms *in vivo* counteracts the anti-cancer effects of CBR agonists (Salazar et al., 2009; Vara et al., 2011). In various cancer types, the cannabinoid-induced autophagy is probably dependent on the p8 molecule, as its signaling is often upregulated upon cannabinoid treatment (Carracedo et al., 2006a,b; Salazar et al., 2009; Vara et al., 2011). Various other regulatory pathways are involved alongside with the p8-mediated autophagy, like an ER stress-dependent upregulation of AMPK coupled with TRIB3-induced downregulation of the AKT–mTORC1 pathway in hepatocellular carcinoma (Vara et al., 2011). In melanoma and breast cancer, activation of CBRs depends also on the downregulation of the AKT pathway (Blázquez et al., 2006; Caffarel et al., 2006), and in gliomas on the activation of TRPV2 receptors (Nabissi et al., 2012; Velasco et al., 2015). When hormone-dependent cancers are taken into account, cannabinoid agonists might also act through cooperation with growth factor-dependent pathways (Guzmán, 2003; Sarfaraz et al., 2008). Thus, the anti-cancer effects of cannabinoids are mediated by various mechanisms depending on the type of cancer cell in question, leading to an autophagy-mediated cell death (Guindon and Hohmann, 2011).

### Regulation of Cancer Angiogenesis and Local and Distant Invasion

It has been reported that the vascular endothelial growth factor (VEGF)-induced cancer cell angiogenesis can be down-regulated by the activation of CBRs in skin carcinomas (Casanova et al., 2003), gliomas (Blázquez et al., 2003, 2004) and thyroid carcinomas (Portella et al., 2003). Additionally, CB1R and/or CB2R agonists lead to an inhibition of adhesion and local and distant invasion in induced and spontaneous metastatic *in vitro* and *in vivo* models of glioma, breast, lung, and cervical cancer (Grimaldi et al., 2006; Blázquez et al., 2008; Preet et al., 2008; Ramer and Hinz, 2008; Qamri et al., 2009). Notably, it has been reported that when ceramide biosynthesis is inhibited *in vitro* or *in vivo*, the anti-cancer and anti-angiogenic effects of CBR activation are abolished (Blázquez et al., 2004).

It is also important to note that CBD acting through non-receptor based mechanisms can generate a significant anti-cancer effect leading to a decrease of the invasive and metastatic potential in various animal cancer models (Velasco et al., 2015).

### Anticancer Immunity Regulation

There are reports that ligand binding to CB2Rs can create a pro-tumorigenic surrounding by interfering with the immune system-mediated tumor surveillance mechanisms (Zhu et al., 2000; McKallip et al., 2005). On the other hand, cannabinoids have also been shown to enhance the tumor surveillance in melanoma xenografts in immunocompetent mice compared to immunodeficient mice, when using a synthetic cannabinoid WIN 55212-2 or JWH-133 (Blázquez et al., 2006; Velasco et al., 2015). Thus, the role of ECS is again dual depending on tumor type and the overall state of the immune system. Additionally, the formation of specific CBR homo- and heterooligomers upon stimulation with cannabinoids, and the subsequent change in their subcellular localization and coupling to G proteins contributes to the complexity of this issue (Velasco et al., 2015).

## Ethical and Legal Aspects of Using ECS Components in Anti-Cancer Therapy

As drug repurposing is significantly faster and more economical than *de novo* introduction of a new drug into the clinic, there is hope that the existing pharmacokinetic and safety data on various ECS components might contribute to their successful translation into oncological healthcare. However, many ethical and legal issues need to be addressed carefully especially before the use exogenous cannabinoids as anti-cancer drugs can become a reality. And while it appears that there are no obvious ethical and legal issues in targeting ECS enzymes/transporters or using endocannabinoids as anti-cancer drugs compared to the issues that exists with exogenous products, the matter of tumor-specificity and adverse effects of endogenous ECS-based approaches needs to be the main research objective.

### Ethical Dilemmas on Using Exogenous Cannabinoids in Medicine

The debate over legalizing medical marijuana started heating up in the United States in the 1970, when the Controlled Substances Act was passed by the Congress. According to this Act, marijuana was listed as a schedule I drug which “has a high potential for abuse, no currently accepted medical use and lacks safety for use under medical supervision.” Being classified under the hallucinogenic substances category, the sale, purchase or consumption of marijuana became illegal.

The debate on whether marijuana can cause addiction and can seriously affect physiological state is still ongoing. Upon smoking cannabis, its active substances rapidly enter the blood stream and are quickly carried to the brain. With regular, heavy use, severe symptoms might arise such as depression, anxiety, irritability, bronchitis, conjunctivitis and endocrine disorders (Volkow et al., 2014). Although animal studies show that it can affect the immune system, there are no studies in humans that definitively correlate the immunosuppressive effects with either increased incidence of infections or immune disorders (Cabral and Staab, 2005). Short term cognitive effects include impairment of short term memory, sensory perception, attention span, problem solving, impaired motor coordination and psychomotor control, distorted judgment, and paranoia and psychosis in cases when higher doses are used (Volkow et al., 2014). It was shown that concurrent smoking of tobacco and marijuana synergistically increases the predisposition to respiratory problems and even chronic obstructive pulmonary disease (COPD). Smoking only marijuana cannot be directly associated with an increased risk of these problematic respiratory states (Tan et al., 2009).

The American Cancer Society (ACS) reported that marijuana’s harmful substances are introduced into the body in a similar manner as tobacco consumption delivers cancer-causing substances. On the other hand, the ACS states that marijuana can alleviate suffering from pain when traditional medicine methods are exhausted. The ACS does not advocate the legalization of marijuana or its use, but they do support more research that would lead to exploitation of its benefits. Mixed reports on the causal relationship of chronic cannabis use and increased risk of cancer are showed in epidemiological literature (Hashibe et al., 2005). Hashibe et al. (2005) found that smoking cannabis was not associated with an increased risk of smoking-related cancers (e.g., lung, head, and neck), but might be associated with an increased risk of prostate cancer, cervical cancer, and glioma. Conversely, Reece reported that smoking cannabis is associated with an increased risk of lung cancer (Reece, 2009). Interestingly, Maertens et al. (2013) found that aside from the cannabinoids and nicotine, cannabis and tobacco smoke condensates contained mixtures that were similar. They also found that cannabis smoke condensate and tobacco smoke condensate influence the same molecular processes but have subtle pathway differences that potentially account for differential toxicities and the mixed results with respect to lung cancer. Even highly controversial, these potential hazards for the individuals seem to be good reasons to justify monitoring and to limit the access to the cannabis use. On the other hand, fear of legal consequences, ethical issues, social and legal barriers, stigma associated with abuse and lack of funding may interfere with the important research on the therapeutic potential of these controversial substances. These impediments could obstruct the research that might not just have the potential to alleviate chronic suffering and exploring harm reduction (Abrams, 1998) but might also have the potential to treat serious illnesses such as cancer.

While deciding on the policies, all harms and benefits of using cannabinoids should be taken into consideration. Also, the harms and benefits that are likely to result from the regulations that limit the access, use and the research on specific drugs must be taken into account. It is required that clinical trials minimize the risks for research subjects and to protect them from unreasonable risk. Reports documenting that cannabinoids reduce pain, nausea and spasms exist (Tramèr et al., 2001; Kalso, 2001), but it has also been argued that these medicinal benefits are overshadowed by the risks and the harmful side effects. There is also the argument of the lack of real need for its use in pain management since many other safer drugs are available (Kalso, 2001). To put more weight on the side of caution, legal and social risks must not be disregarded. Based on its unpopular and stigmatized use mostly in the Western countries, it seems that decisive clarity on whether benefits outweigh the risks of cannabinoid use for medical purposes will not be reached in the near future. When professionals’ opinions are taken into consideration, it seems that the majority of medical doctors are in favor of medical marijuana use.

In this ongoing debate, the main arguments for allowing cannabis use come from the ideas of autonomy and freedom. Although the notion of autonomy has been used in many different relations, in biomedical ethics the idea of autonomy actually means self-government. The point of view that the value of patients’ autonomy is more important than the value of protecting the patients’ well-being highlights the bioethical issues that arise in the context of clinical research (Varelius, 2006). The definition of autonomy most often used in medical ethics is derived from the philosophy of Immanuel Kant who proposed that autonomy means personal self-determination. According to his doctrine, people can act “truly free” only if they act to their desires, attitudes or emotions. One could argue that cannabis use should not be forbidden to anyone, especially since there is a valid medical basis for its use. However, one could also argue that the use of psychoactive substances leads to addiction and clouds a person’s judgment which seriously confronts the Kantian model since addiction is prone to hinder self-determined decision making.

The formal bioethical principle of justice requires treating similar cases alike and different case differently. This concept also emphasizes fairness and equality among individuals. Since marijuana is listed as harmful and addictive and is banned as a class I drug, it can be stated that other harmful drugs as nicotine in the form of cigarettes and alcohol should be regarded alike and should not be allowed for use. The main question involving social justice can be formulated in the following manner: is it just to allow the research of substances that may alleviate conditions in one patient and prohibit or limit research on substances that might be the only ones that can help another patient? If the answer is yes, additional questions arise. When should such research be limited, why and by whom? Who is to decide about the fairness of the research? (Andreae et al., 2016). Respecting the autonomy and following the notion of social justice it does appear that marijuana should be legalized for medicinal use from the ethical point of view. Legalization of marijuana would be an example of utilitarianism if the positive outcome of such an action would outweigh the negative consequences. In case we consider drug use not only for symptoms alleviation but for the treatment of the disease itself, it seems that this risk/benefit calculation may be favorable in terms of greater benefits. While assessing physical risks we must not forget the psychological, social, legal risks and risks regarding privacy issues. Also, many risks vocalized through the media tend to be exaggerated and may distort our judgment of the real risks.

### Legalization Issues

In June 2018, Canada made history by passing the Bill C-45 which is officially known as the Cannabis Act. By doing this, Canada has become the first industrialized nation in the world to have passed legislation allowing adults to purchase marijuana for recreational use. The only country in the world that has similar legislation so far is Uruguay. Per the Canadian law, adults over the age of 18 are allowed to purchase marijuana, as well as possess up to 30 g of dried cannabis for personal consumption (Canada Gov, 2018; CTV, 2018). Sale to minors is strictly prohibited.

Aside from Canada, USA’s Vermont became the first US state to allow use of recreational cannabis entirely through the legislative process in the beginning of 2018. Meanwhile, Oklahoma passed legislation that legalized medical marijuana. Even though their approaches might be slightly different, 30 states in the United States allow marijuana for medical purpose (“Marijuana Is Legal for Medical Purposes in 32 States – Vox” 2018). This changing perception toward cannabis has been happening almost everywhere in the world and a growing number of countries have legalized medical marijuana to some extent. The use of marijuana in the medical setting has been fully legalized in Czechia. The Netherlands has also legalized its use and restricted it to “coffee shops,” while outside of coffee shops, it is considered illegal (although decriminalized) to possess a maximum of 5 g or 5 plants. Poland, Romania, Norway, Germany, Italy, Greece have all legalized access for medical use. There are some European countries where cannabis is not legalized for smoking such as France, Spain and Slovenia but the use of cannabis-derived drugs is permitted. In Israel, Romania, Macedonia and Puerto Rico, marijuana use is illegal, but has been made available in cases of severe or terminal illnesses. Following the examples of Uruguay, nine states in the United States and most recently Canada, there have been many initiatives to propose legalizing cannabis in the more restrictive counties. For example, Spain has reignited the debate over legalizing marijuana. In Australia medical marijuana is legal at the federal level but the implementation is not the same in all states. In Victoria and New South Wales a cannabis card is necessary, and in the Australian Capital Territory and South Australia personal cultivation of up to two plants is allowed.

According to the UN survey (CNN, 2018) over 10,000 tons of cannabis is produced in the African continent each year and Africa appears to be the next large market for cannabis legalization. In May 2018, Zimbabwe legalized growing marijuana for medicinal and research purposes. Similarly, Lesotho began approving medical marijuana culturing licenses in 2017. The UN Office on Drugs and Crime reported that Morocco is the second-largest producer of cannabis in the world. Because of the huge potential that this industry could have on Morocco’s economy, in 2014 the bill to legalize marijuana production for medical and industrial use was proposed in the Moroccan parliament. The bill failed at the time due to political and religious reasons and it seems that it will be difficult to overcome them any time soon.

## Clinical Trials

Clinical trials have shown important findings investigating various components of the ECS (Table 2). FAAH is a membrane enzyme that degrades anandamide and is therefore a very attractive pharmacological target in many diseases. Many efforts have been employed to design and test new FAAH inhibitors or modulators of its activity (Lodola et al., 2015). However, the very unfavorable outcome of the French phase I study using the BIA 10-2474 FAAH inhibitor on healthy human volunteers is a dramatic example of how unpredictable the translation of ECS-based drugs into the clinic can be (Kaur et al., 2016). Currently, there are no approved FAAH inhibitors in clinical use, although the US FDA (in collaboration with the European Medicines Agency and the French national medicines agency) released a statement after the failure of this trial that other classes of FAAH inhibitors do not pose similar safety risks (FDA, 2016).

**Table 2:** Clinical trials involving the endocannabinoid system in the period 2009–2018.

Study name	Year Country	ECS target component	Objective or outcome	References
Titration Study of ABX-1431	2018 Belgium	MAGL inhibitor	Assessment of safety, tolerability, pharmacodynamics and pharmacokinetics	https://clinicaltrials.gov/ct2/show/NCT03447756
Pilot trial assessing efficacy and safety of medicinal cannabis in patients with gliomas	2016 Australia	Exocannabinoid	Assessment of progression-free survival	https://www.endeavour.edu.au/about-us/news/impact-of-medicinal-cannabis-on-australians-with-malignant-brain-tumours
A double-blind, randomized, placebo-controlled, combined single and multiple ascending dose study of BIA 10-2474	2015 France	FAAH inhibitor	One exitus, five volunteers hospitalized with neurological side-effects	https://www.youscribe.com/BookReader/Index/2691486/?documentId=2855144
Report of Objective Clinical Responses of Cancer Patients to Pharmaceutical-grade Synthetic Cannabidiol	2015 United Kingdom	Exocannabinoid	Reduction in circulating tumor cells/reduction in tumor size	Anticancer Res. 2018, 38(10):5831–5835
A Safety Study of Sativex Compared With Placebo (Both With Dose-intense Temozolomide) in Recurrent Glioblastoma Patients	2014 United Kingdom and Germany	Exocannabinoid	Improved survival rate	https://clinicaltrials.gov/ct2/show/NCT01812616
A study of a 1:1 ratio of the cannabinoids cannabidiol and delta-9-tetrahydrocannabinol (CBD:THC) plus dose-intense temozolomide in patients with recurrent glioblastoma multiforme	2013 United Kingdom	Exocannabinoid	Some efficacy in patients with recurrent GBM as an adjunct to dose-intense temozolomide	J Clinical Oncol, 2017, 35(15)suppl:2046
Dexanabinol in Patients With Brain Cancer	2012 United States	Exocannabinoid	Assess the effect on tumor size	https://clinicaltrials.gov/ct2/show/NCT01654497
A Phase 1 Study of Dexanabinol in Patients With Advanced Solid Tumors	2011 United Kingdom	Exocannabinoid	Asses the reduction in size of tumor(s)	https://clinicaltrials.gov/ct2/show/study/NCT01489826
A population-based case-control study of marijuana use and head and neck squamous cell carcinoma	2009 United States	Exocannabinoid	Moderate marijuana use is associated with reduced risk of HNSCC	Cancer Prev Res (Phila Pa). 2009, 2(8):759–68


Because of its key role in the degradation of the 2-AG, targeting MAGL represents an interesting therapeutic target. In addition, MAGL is over-expressed in various cancers (breast, ovarian, melanoma) thus its inhibition can lead to a decrease of migration of cells and their invasiveness (Granchi et al., 2017). Also, MAGL controls the release of fatty acids from lipid-rich cancer cell compartments, which can lead to an activation of lipid signaling pathways implicated in migration, invasion, survival, and tumor growth. Thus, MAGL inhibitors have proven as promising candidates for anti-cancer therapy. Because of its specific properties, many compounds with a MAGL inhibition activity were investigated by academia and pharmaceutical companies. MAGL inhibitors have been patented for a large number of therapeutic uses, mainly covering the area of pain and inflammation, metabolic disorders (such as obesity and diabetes), neurodegenerative pathologies (such as Alzheimer’s disease), as well as the treatment of cancer, anxiety, and epilepsy (Granchi et al., 2017).

Following the example of Ben Amar who reviewed the existing clinical trials using cannabis and exogenous cannabinoids from 1975 to June 2005 (Ben Amar, 2006), two review papers were issued in 2010 and in 2014 reporting more recent clinical trial data. Both of these reviews were based on systematic research of PubMed for published randomized (double) blinded, placebo-controlled clinical trials, using the following keywords: cannabis, marijuana, marihuana, hashish, cannabinoid(s), tetrahydrocannabinol, THC, CBD, dronabinol, Marinol, nabilone, Cannador, nabiximols and Sativex.

In the review that covered period between 2006 and 2010, 37 such studies were identified (Hazekamp and Grotenhermen, 2010a), and 8 main indications of cannabinoid use were: neuropathic or chronic pain, experimental pain, multiple sclerosis and spasticity, HIV/AIDS, glaucoma, intestinal dysfunction, nausea/vomiting/appetite and schizophrenia. Based on the data presented in this study, cannabinoids showed most promise for the treatment of multiple sclerosis, but also as analgesics in chronic neuropathic pain and as appetite stimulants in cancer and AIDS. A wide range of cannabis-based drugs exhibited analgesic effects on various types of chronic and neuropathic pain with the majority of the adverse effects being mild or moderate, while they did not have such a prominent effect on acute types of pain.

One of the first studies performed to evaluate cannabinoid antitumoral action was performed by Guzmán and collaborators, who showed that cannabinoids can inhibit tumor growth (Guzmán, 2003). Due to the ethical and legal issues discussed previously, the first studies were conducted in terminal patients with recurrent tumors (Guzmán et al., 2006). These first trials shed the light not just on the palliative effects, but also on the possible antitumoral effects of cannabinoids, alone or in combination with other drugs as well.

In the review that covered the period between 2010 and 2014, 32 controlled studies were reported that further investigated the therapeutic potential of cannabinoids (Hazekamp and Grotenhermen, 2010b). Eleven main indications for which cannabinoids showed greatest treatment potential included: chronic pain, multiple sclerosis, irritable bowel syndrome, Crohn’s disease, appetite and chemosensory perception, chemotherapy-induced nausea and vomiting, pulmonary diseases, cannabis dependence, psychosis and Parkinson’s disease. Compared to the previous report period, the effect of oral cannabis on patients suffering from chronic pain was much more investigated, as there was an increase in the number of patients participating in pain-related clinical trials. Because of the various limitations of cannabinoid use in a clinical setting for most of the investigated conditions, chronic pain currently remains the major field of research interests as it is linked to less controversy while providing an acceptable benefit.

There are many review papers and meta-analyses discussing the safety, toxicology and therapeutic effects of exogenous cannabinoids. Out of the 70 known cannabinoids derived from the plant Cannabis sp., variety of synthetic cannabinoids and cannabinoid extracts, THC and CBD are the most commonly researched cannabinoids in the literature. Different methods of administration, inconsistent dosing measures, and highly variable cannabinoid content of cannabis plants only add to the already complex interactions of exogenous and endogenous cannabionids. Factors such as light, temperature, humidity, and soil type during cultivation, genetic factors, the method of administration (e.g., oral, smoked, vaporized) and form of cannabinoid consumed (e.g., stems and buds, hashish, hash oil, extract, synthetic) can impact the response to use. Since future clinical trials focusing on anticancer effects of cannabinoids will most likely not be conducted as monotherapy, research studies concerning probable interactions between cannabinoids and cytostatic drugs have shown promising effects. For example, THC and CBD enhance the cytotoxic impact of several chemotherapeutics such as cytarabine, doxorubicin, mitoxantrone, carmustine, temozolomide, bortezomib, carfilzomib and cisplatin (Ramer and Hinz, 2017). THC and CBD have been also shown to enhance the cytostatic effect of vinblastine in resistant leukemia cells and of mitoxantrone in embryonic fibroblasts (Holland et al., 2006, 2007). An enhancement of the cytostatic properties of cytarabine, doxorubicin and vincristine has, likewise, been substantiated for THC in leukemia cells (Liu et al., 2008). Synergistic actions were further reported for the effect of a CBD/THC combination added to multiple myeloma cells in the presence of carfilzomib. The susceptibility of glioblastoma cells for the cytotoxic action of cisplatin was found to be enhanced by CBD (Deng et al., 2017).

In 2017, phase II, randomized, placebo-controlled clinical trial with recurrent glioblastoma multiforme patients was announced and showed the potential efficacy of cannabinoids as add-on anti-cancer drugs. 12 patients were randomized to a combination of THC and CBD in addition to dose-intensive temozolomide, whereas 9 patients were randomized to placebo plus standard of care. This study showed a significantly higher one year survival rate in the cannabinoid group (83% vs. 53%), and the median survival for the cannabinoid group was greater than 550 days comparing to 369 days in the placebo group (GW Pharmaceuticals, 2017 press release; ClinicalTrials.gov Identifiers: NCT01812616, NCT01812603).

## Future Directions

Although the ECS has been studied in detail by many research groups for decades, its clinical value as an anti-cancer target is still under debate. More intensive basic research is needed for a more precise characterization of the biochemical ECS mechanisms that are crucial in the cancer-setting, especially when cannabinoid-based/standard drug combinations are taken into account. Rigorous testing before any such drug should advance to phase-I clinical trials needs to be a matter of global consensus. Proper selection of target patient groups needs to be intensified to comply with all Good Clinical Practice regulations, as well as bioethical principles and legal boundaries, in order to achieve the right drug response in the right patient at the right moment.

## Conclusion

There is an overwhelming burden of evidence that the ECS and all its components is an attractive anti-cancer target, but different strategies are yet to be rigorously clinically tested in order to exploit its full potential, such as explore the value of cannabinoid receptor heteromers as potential new targets for anti-cancer therapies and as prognostic biomarkers. Taking into account all the ethical issues involved in the use of ECS exogenous ligands in anti-cancer therapy and the number of ongoing clinical trials, we are definitely still not there yet, but the route is firm and sprinkled with hope for success.

## Author Contributions

All authors wrote the manuscript, contributed to manuscript revision, and read and approved the submitted version.

## Conflict of Interest Statement

The authors declare that the research was conducted in the absence of any commercial or financial relationships that could be construed as a potential conflict of interest.
